# Mechanisms of Population Structuring in Giant Australian Cuttlefish *Sepia apama*

**DOI:** 10.1371/journal.pone.0058694

**Published:** 2013-03-11

**Authors:** Nicholas L. Payne, Edward P. Snelling, Jayson M. Semmens, Bronwyn M. Gillanders

**Affiliations:** 1 School of Earth and Environmental Sciences, University of Adelaide, Adelaide, South Australia, Australia; 2 School of Biological, Earth and Environmental Sciences, University of New South Wales, Kensington, New South Wales, Australia; 3 Institute for Marine and Antarctic Studies, University of Tasmania, Hobart, Tasmania, Australia; University of Bologna, Italy

## Abstract

While a suite of approaches have been developed to describe the scale, rate and spatial structure of exchange among populations, a lack of mechanistic understanding will invariably compromise predictions of population-level responses to ecosystem modification. In this study, we measured the energetics and sustained swimming capacity of giant Australian cuttlefish *Sepia apama* and combined these data with information on the life-history strategy, behaviour and circulation patterns experienced by the species to predict scales of connectivity throughout parts of their range. The swimming capacity of adult and juvenile *S. apama* was poor compared to most other cephalopods, with most individuals incapable of maintaining swimming above 15 cm s^−1^. Our estimate of optimal swimming speed (6–7 cm s^−1^) and dispersal potential were consistent with the observed fine-scale population structure of the species. By comparing observed and predicted population connectivity, we identified several mechanisms that are likely to have driven fine-scale population structure in this species, which will assist in the interpretation of future population declines.

## Introduction

Defining the scale of exchange among populations and the factors driving this exchange underpin our understanding of the dynamics, genetic structure, and biogeography of aquatic animals [1]. The life-histories of most marine species include at least one widely dispersive stage, and the scale of this dispersal is one of the primary determinants of population structure [2]. Broad dispersers are genetically homogenous over larger spatial scales, and their ability to adapt to local conditions can be compromised [3], whereas local retention of larvae by behavioral or oceanographic mechanisms drives greater local adaptation and genetic differentiation [3]. As such, identifying the mechanisms that facilitate broad versus local dispersal is considered a major challenge facing marine ecologists and managers [4], [5].

The importance of describing marine connectivity has led to the development of a wide range of approaches, including genetic or morphological comparisons [3], [6], examination of otolith chemistry [7], [8] and external or chemical tagging techniques [9], [10]. However, identifying the mechanisms that drive population structuring is exceedingly difficult, and a poor understanding of factors driving differentiation hinders the accurate prediction of a population’s response to environmental change. If however, a comparison can be made between observed population connectivity and that which is predicted (based upon known life-history strategies, potential for dispersal, and oceanography experienced by a species, etc.), mechanisms driving structure can be better understood, and this will increase the ability to predict a population’s response to change.

Large-scale movement has been observed in all stages of cephalopod life history [11], from passive drifting of paralarvae to migrations over several thousands of kilometres in adults [12]. Together with oceanographic currents [13], [14], the swimming capacity of cephalopods is a major determinant of population connectivity, and cephalopods exhibit significant variability in swimming performance. For example, the northern shortfin squid *Illex illecebrosus* can sustain swimming speeds in excess of 1 m s^−1^ [15], with extensive migrations in this species [16] facilitated by optimal cost of transport (COT_opt_) speeds in the region of 0.6 m s^−1^ [17]. At the other end of the cephalopod spectrum, maximum aerobic performance occurs at less than 0.2 m s^−1^ in the chambered nautilus [17], with tracked animals preferring speeds of less than 0.05 m s^−1^ [18]. Clearly swimming capacity represents a major variable for identifying mechanisms driving differentiation (or connectivity) among cephalopod populations.

**Figure 1:**
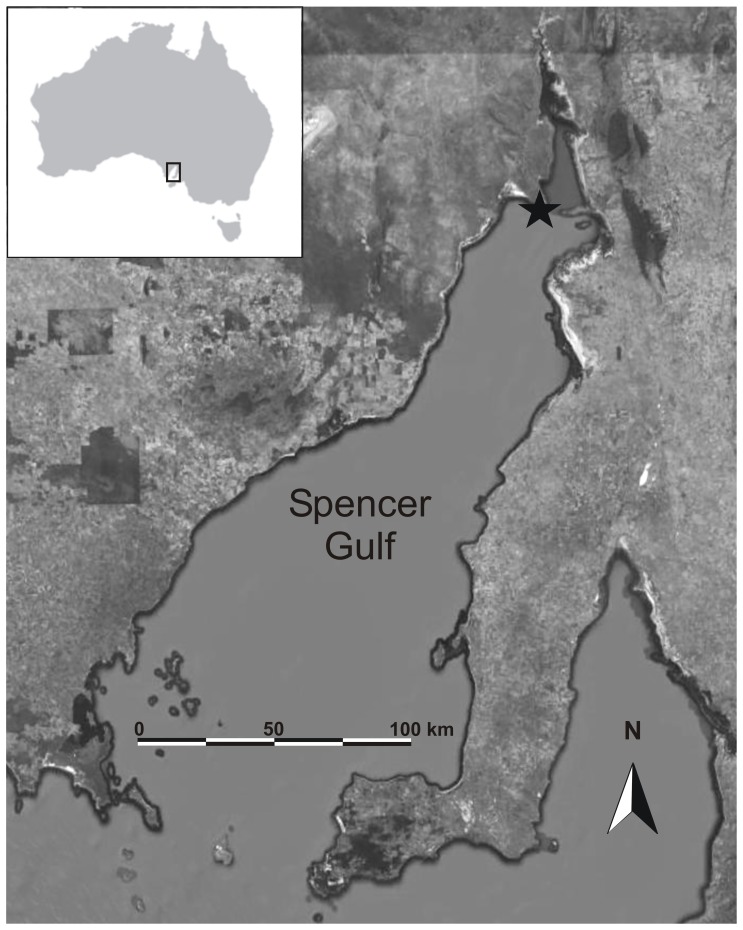
Location of *Sepia apama* breeding aggregation (black star) in northern Spencer Gulf, South Australia. Image source: Google Earth mapping service.

*Sepia apama* is the largest cuttlefish species known, is endemic to the temperate and sub-tropical waters of southern Australia, and forms the only-known dense spawning aggregation of cuttlefish. From May to August each year, hundreds of thousands of mature *S. apama* converge on the small (∼ 60 ha) strip of rocky reef at Point Lowly, South Australia (33.00°S, 137.75°E; Fig. 1) to breed, and a strongly male-biased operational sex ratio has led to the development of spectacular mating behaviors [19]–[22]. Like most other cephalopods, *S. apama* are short-lived (12–-24 months [23]) and semelparous, spawning once at the end of their life-cycle. Given recent declines in spawning aggregation biomass [24], understanding the degree of connectivity between the Point Lowly aggregation and other parts of its range will be critical for predicting population viability. It has been suggested that at least three distinct populations are likely in South Australia [6], and preliminary microsatellite, morphological and statolith chemistry data suggests very little mixing of individuals over scales as small as 100 km (B.M. Gillanders unpublished data). While several aspects of *S. apama* biology and ecology have received attention [19], [21], [25]–[29], a major hindrance to identifying mechanisms driving fine-scale structuring is a poor understanding of the sustained swimming capacity (a measure of dispersal potential) of *S. apama.*

The aim of this study was to describe the energetics and sustained swimming capacity of *S. apama.* By combining this information with our understanding of the life-history strategy, behaviour and oceanography experienced by *S. apama*, our objective was to predict the scale of connectivity between sub-populations of this species, and compare these predictions to preliminary genetic data on *S. apama* population structure. In doing so, we hope to achieve a better mechanistic understanding of population differentiation in this species.

## Materials and Methods

### Ethics statement

All necessary permits were obtained for the described field studies (Primary Industries and Resources SA S115 ministerial exemption # 99022244), and all research was conducted with approval from the University of Adelaide Animal Ethics Committee (# S-047-2007A). The location is not privately-owned or protected in any way, and the field studies did not involve endangered or protected species.

### Study species

*Sepia apama* (*n*  =  25 [1 female, 24 male]; 297–995 g wet mass; 15–22 cm mantle length) were collected via SCUBA (cuttlefish were generally preoccupied with breeding so could readily be captured by hand) from breeding grounds at Point Lowly, South Australia (33°00’S, 137°44’E), during July and August 2009. Each cuttlefish was immediately placed into individual, aerated, 68-L plastic tubs, before being transported to the University of Adelaide, South Australia (34°92’S, 138°60’E). Tubs were constantly aerated during the 5 h transportation, and a 75% water change was conducted twice en route. Immediately upon arrival, each cuttlefish was transferred to individual aquaria (500-L) housed within a 14 ± 1°C controlled temperature room (equivalent to temperature at Point Lowly aggregation site in July-August). 75% water changes were performed daily for each aquarium for the period in which cuttlefish were maintained prior to swim trials. All experiments were performed within 72 h of collection, and each individual was fasted for the entire 72 h prior to swim trials to minimise oxygen uptake due to feeding and digestion.

### Respirometry, finning and jetting frequency

An 80-L Brett-type swim tunnel respirometer was used to manipulate *S. apama* swimming speeds and to measure metabolic rates. The perspex swim chamber was 750 mm long, 200 mm wide and 107 mm deep, and the centre section of the lid was constructed of transparent plastic film. Prior to measurements, each cuttlefish was acclimated to the swim tunnel for 30 min, during which time individuals settled in the upstream-half of the swim chamber (covered with dark black plastic) and displayed only occasional finning. Resting oxygen consumption rates were recorded for 30 min, immediately followed by measurements of oxygen uptake during swimming at a randomly-selected speed - 0.7, 11, 15 or 25 cm s^−1^. Five individuals were swum at each speed, and five were used only to quantify resting oxygen consumption rate. Cuttlefish were allowed to choose their preferred swimming orientation, and the swim chamber was sufficiently wide for individuals to change orientation during swimming trials (only one individual was greater than 19 cm ML, and the plastic film in the lid could be pushed outward several cm when cuttlefish turned). Each swim trial lasted 180 min (considered to be sustained aerobic exercise in fishes [30]–[32]) or until cuttlefish fatigued, which was defined as an unwillingness to return to the upstream-half of the swim chamber following 10 s of prodding of the arms with a blunt probe. In these instances, time until fatigue was recorded. Immediately following trials, wet body mass and mantle length, width and height were recorded. Some cuttlefish occupied > 10% of the cross-sectional area of the swim chamber, so a blocking correction was applied to all swimming speeds to account for the increased water speed caused by the profile of the animal [21], [33]–[35].

All swim trials were recorded on a digital video camera (HDC-SD1, Panasonic, Japan) that was positioned adjacent to the swim chamber. The number of complete fin undulations and propulsive jets were counted during three, randomly-selected, 1 min intervals during resting and swimming trials for each cuttlefish. Mean finning frequency and jetting frequency were then calculated for resting cuttlefish and swimming cuttlefish at 7, 11, 15 and 25 cm s^−1^.

As a preliminary examination of the sustained swimming capacity of the early life-history stage of *S. apama*, seven eggs were collected from the aggregation area in June 2009 and transported to the University of Adelaide aquaria. Within 48 h of hatching, each cuttlefish (12.3 ± 0.4 mm ML; mean ± SE) was placed in a 1-L swim chamber (10 cm cube) made from 3×3 mm wire mesh, which was positioned inside the working section of the Brett-type swim tunnel used in the adult experiments. Water velocity was immediately increased to 3–4 cm s^−1^, which was the lowest velocity that could be maintained consistently with our flume design, and is similar to the current speeds the hatchlings are likely to experience at Point Lowly in winter (J. Kaempf, personal communication). Time to fatigue was measured using the same protocol as for adults.

### Data analyses

Oxygen consumption was measured with a polarographic probe (YSI, Yellow Springs, Ohio. Model 58 meter and 5739 electrode) that was ventilated at a constant rate (with an external pump in a separate circuit) independent of flume velocity. The probe was calibrated daily by oxygen-saturated air in the aquaria. Oxygen consumption rate (V^.^ o_2:_ ml kg^−1^ h^−1^) was calculated for each cuttlefish at each exercise intensity from the decline in dissolved oxygen content over time, given 5.89 ml O_2_ L^−1^ in oxygen saturated saltwater (38 g L^−1^) at 14°C [36]. Least-squares regression was used to estimate the relationship between swimming speed (cm s^−1^) and oxygen consumption rate, and between finning frequency (Hz) and jetting frequency (Hz) using data from all 25 cuttlefish. Analyses were undertaken with Sigmaplot 10.0 (Systat Software, Inc). Gross aerobic metabolic cost of transport (GCOT; J kg^−1^m^−1^) was calculated by converting rates of oxygen consumed to joules of energy expended (assuming 1 ml of oxygen  =  20 J [37]), and dividing these values by their corresponding swimming speeds.

## Results

### Respirometry and fatigue tests

**Figure 2:**
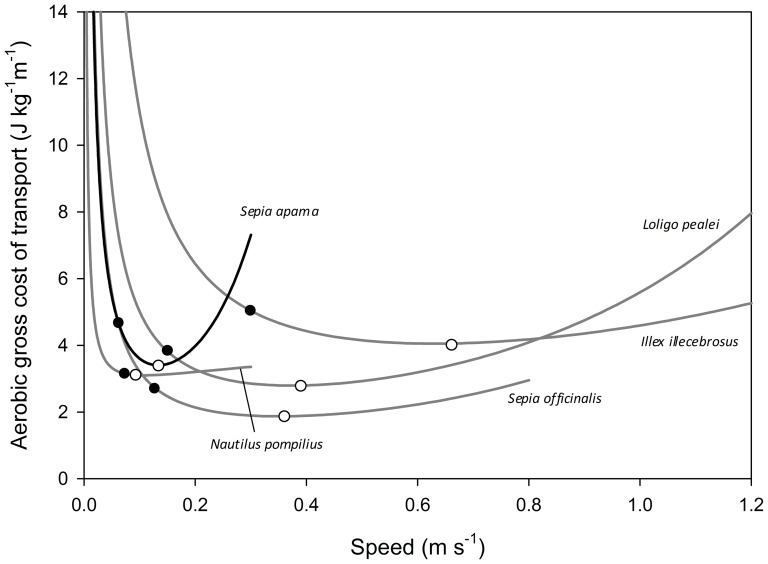
Estimated gross cost of transport in cephalopods, including the minimum cost (open circles) and actual tracked speeds (filled circles). Data for *S. apama* are derived from Equation 1 and tracked speeds in the field [21]. Data for other species are from O’Dor & Webber [17] and O’Dor [39].

A description of the relationship between swimming speed and V^.^ o_2_ is provided in [21]. Briefly, V^.^ o_2_ increased in an exponential manner from resting (40.8 ± 2.2 ml kg^−1^ h^−1^) up to 15–16 cm s^−1^ (97.1 ± 5.0 ml kg^−1^ h^−1^), which appeared to represent the maximum aerobic speed for *S. apama* (there was a slight reduction in mean oxygen consumption rate above this speed [21]). The 25–27 cm s^−1^ treatment was associated with a rapid onset of fatigue (see below), indicating these higher speeds were partly supported by anaerobic metabolism. We therefore excluded oxygen consumption measurements from the 25–27 cm s^−1^ treatment from our regression analyses. The increase in oxygen consumption rate with swimming speed (*U;* cm s^−1^) up to 16 cm s^−1^ is best described by the exponential model:
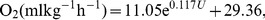
(1)


and from this equation, we estimated the minimum aerobic gross cost of transport as 3.40 J kg^−1^m^−1^, at a speed of 13.4 cm s^−1^ (Fig. 2).

**Figure 3:**
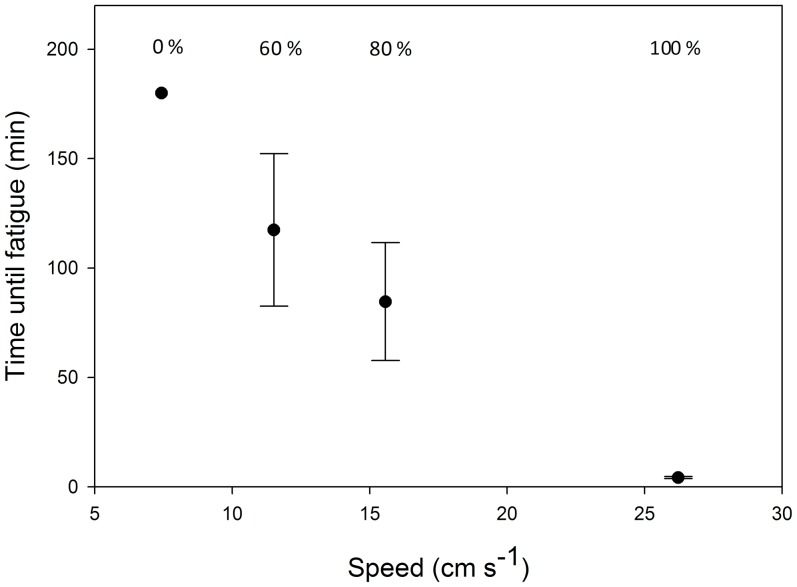
Time taken to fatigue (mean ± SE) for *S. apama.* Five individuals were swum at each speed, and the percentage of individuals that fatigued within 180 min of swimming is indicated.

All individuals swum at 7–8 cm s^−1^ were able to maintain steady-state swimming for the entire 180 min trials without succumbing to fatigue (Fig. 3). However, higher swimming speeds led to increased incidence of fatigue, and a decrease in the mean time cuttlefish could sustain swimming (Fig. 3). At the highest swimming speed, all five individuals fatigued within 5 min (mean 4.2 ± 0.5 min SE).

For hatchlings, the maximum time until fatigue was 10.0 min, although most fatigue times were far lower (mean 4.7 ± 1.1 min), and these were continuously supported by mantle jetting throughout the duration of all trials. Linear regression did not detect a significant relationship between mantle length and time to fatigue for hatchlings (*P* > 0.05).

### Finning and jetting frequency

**Figure 4:**
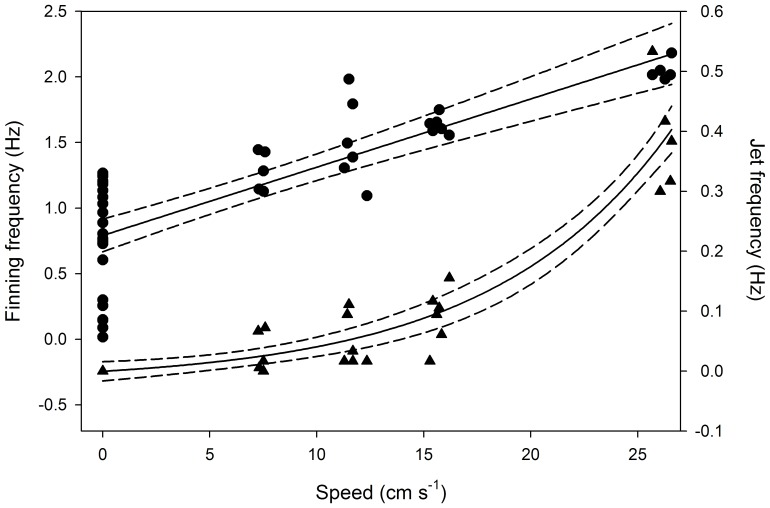
Relationships between swimming speed and frequency of finning (circles; see equation 2 in text) and propulsive jets (triangles; equation 3 in text) for giant Australian cuttlefish *Sepia apama*. Dashed lines represent 95% confidence intervals.



Finning frequency (ƒ_f_ ; Hz) increased linearly with swimming speed (*U*; cm s^−1^; *P* < 0.0001; *R* ^2^  =  0.68; Fig. 4), following the equation:

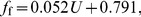
(2)whereas the increase in jetting frequency (ƒ_j_ ; Hz), with swimming speed (*P* < 0.0001; *R* ^2^  =  0.90; Fig. 4) was best approximated by the exponential model:
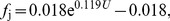
(3)


such that low swimming values were supported primarily by finning, and the frequency of propulsive jets increased considerably at the highest swimming speed (25–27 cm s^−1^).

## Discussion

### Swimming energetics of *Sepia apama*

Our estimate of the speed that minimises aerobic cost of transport is approximately one third (or less) of that estimated for several squid and cuttlefish species, with both predicted speeds and those measured in the field more similar to those of the other buoyancy-regulating group of cephalopods, the chambered nautilus ([18] Fig. 2). The relatively narrow range of speeds over which swimming could be maintained (< 25 cm s^−1^) likely drives the more rapid increase in COT above that of COT_opt_ than for other cephalopods, and this higher section of the *S. apama* swimming spectrum was associated with a transition from fin undulations to propulsive jetting - a mode of transport typically reserved for squid, or for short bursts of activity associated with predator avoidance in cuttlefish [14]. Not only is our estimate of COT_opt_ achieved at a low swimming speed, but 50% of individuals swum at between 11 and 16 cm s^−1^ (close to our estimated COT_opt_; 13.4 cm s^−1^) could not sustain swimming beyond 90 min, and 70% of individuals had fatigued within 180 min of steady-state swimming. We did not measure octopine (the major by-product of anaerobic glycolysis in cuttlefish [38]), but an anaerobic contribution to locomotion would significantly increase the gross COT during swimming at 11–16 cm s^−1^, which would further reduce the speed at which COT_opt_ is achieved. The range of speeds over which *S. officinalis* from O’Dor & Webber [17] were swum is unclear, so whether the far greater speed of COT_opt_ for that species (more than double our estimate) is a function of extrapolation from slower speeds or direct measurement at those speeds is uncertain. The condition of our cuttlefish (midway through a semelparous, protein catabolism-fuelled spawning event) may have been somewhat less than cuttlefish earlier in the spawning season, and this may explain some of the differences in swimming performance between the two *Sepia* species. Clearly the marked difference in optimal swimming speeds for these two species of similar size, morphology and life-history warrants further investigation.

Given that all speeds above 7–8 cm s^−1^ led to a high prevalence of fatigue, we consider it likely that the observed sustained daytime speeds (6.0 ± 2.1 cm s^−1^; mean ± standard error of estimate) of free-ranging *S. apama* [21] approximate the maximum sustainable speeds for this species. This interpretation is in agreement with data on free-ranging and laboratory-reared *S. apama* from other studies ([39], [40] respectively). Interestingly, previous respirometry data from three *S. apama* individuals reared in captivity and in warmer (20–21°C versus 14°C from the present study) water [40] suggest resting and maximum oxygen consumption rates approximately two- to three-fold higher than from our study, resulting in a *Q*_10_ (the increase in metabolic rate caused by a 10°C increase in temperature) between 3 and 6. The *Q*_10_ of cephalopods is generally close to 2 [37], [41], [42], and while the smaller mean body mass of individuals from the earlier study (approximately half that of cuttlefish used in the present study) might partly explain their higher mass-specific metabolic rates, identifying the remaining sources of metabolic rate variation in captive versus wild-sourced cuttlefish could be a rewarding avenue for future study.

### Mechanisms of self-recruitment

While the population structure of *S. apama* conforms to a traditional model of isolation-by-distance across most of its range [6], microsatellite, morphological and statolith chemistry data recently demonstrated clear population divergence in *S. apama* approximately 100 km south of the Point Lowly aggregation site (B.M. Gillanders unpublished data). Identifying the mechanisms driving this striking lack of gene-flow is particularly critical in light of the significant declines in biomass observed at the Point Lowly aggregation over the past decade [24].

During January and February, *S. apama* are immature and feeding in northern Spencer Gulf, whereas from May–August, mature cuttlefish at the aggregation site are not feeding [19], [25]. If the onset of a spawning migration coincides with that of maturity, then cuttlefish must complete their migration within 2 months if they are to reach the aggregation site by the start of the spawning period (May). *Sepia apama* are diurnally active, resting on the bottom at night (at least during spawning [20], [21], and non-breeding *S. apama* also appear to be diurnally active [43]), so a 2 month migration at 6–7 cm s^−1^ during daylight hours could cover approximately 140 km if unassisted by currents. Spawning *S. apama* are thought to mobilise approximately 30% of their tissue to fuel reproduction prior to death, at a rate of 0.8% body mass d^−1^ [21], [27], however, summer water temperatures in northern Spencer Gulf are up to 10°C higher than in winter. These higher temperatures would increase the rate of daily energy expenditure to some extent, such that a 60 d (140 km) migration, at a rate of > 0.8% body mass loss per day, could probably not be supported by energy reserves alone. Although we do not have estimates of feeding success for wild *S. apama*, it is likely that refueling requirements (as proposed by [39], [40]) would reduce the distance they could swim in 60 days to somewhat less than 140 km, a prediction consistent with the approximate distance between Point Lowly and the barrier of genetic divergence to the south (100 km; B.M. Gillanders unpublished data). Passive northward advection via currents and selective tidal-stream transport (STST) would increase migration distances, both of which are possible. However, density-driven circulation in Spencer Gulf is weakest in summer [26], [44], and recent hydrographic modelling suggests almost 90% of passive particles released throughout Spencer Gulf in February that are in the vicinity of Point Lowly by June originated in northern Spencer Gulf. This suggests *S. apama* are unlikely to get a ‘free ride’ to Point Lowly from southern Spencer Gulf unless they employ STST (testing for STST in *S. apama* and other cephalopods may prove feasible with current electronic tagging techniques).

**Figure 5:**
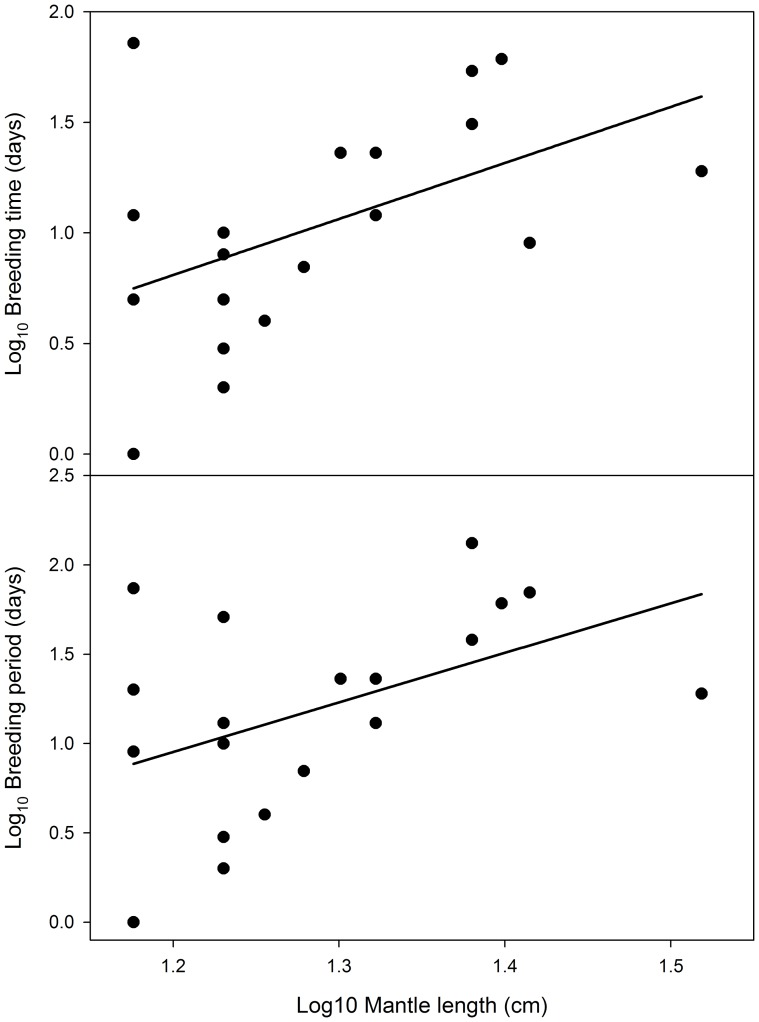
Relationships between *S. apama* size and breeding durations using data in Payne et al. [27]. Breeding time is the number of days each individual was present at the Point Lowly breeding aggregation, and breeding period is the number of days between the first and last day that each individual was present. Least-squares regression identified a significant effect of size for both metrics (*n*  =  19; *P* < 0.05, *r* ^2^  =  0.24 and 0.22 for breeding time and duration, respectively).

It is possible that individuals hatching in southern Spencer Gulf begin a northward migration earlier than late February, however given evidence for synchronous spawning throughout Spencer Gulf [25], such a migration would need to begin at approximately 2–3 months of age, and would therefore require significant growth and maturation to occur *en route*. Furthermore, with a rapid onset of fatigue for hatchlings swum at just 3–4 cm s^−1^, the early stages of such a migration are likely to occur at a much slower pace. There is evidence for a class of longer-lived (2 years), larger-bodied *S. apama* in Spencer Gulf [23]; animals that would not have the migration-time constraints of the shorter-lived cohort. However, while the spawning aggregation is comprised of similar densities for both 1- and 2-year-old cuttlefish [23], the majority (70–80%) of *S. apama* throughout broader Spencer Gulf belong to the former class, and an analysis of data from Payne et al. ([27] data were re-analysed to compare breeding durations with size, rather than sex as per the previous publication) suggests an increase in breeding durations with size at Point Lowly (Fig. 5). Such a disparity would lead to an overestimation of relative abundance of the 2-year class at the breeding aggregation, so we consider it likely that most *S. apama* in Spencer Gulf have a life-cycle of approximately 12-months, and that the similar densities of both classes at the aggregation is a result of extended residence times for the larger, slower-growing individuals. Only low levels of gene flow are required to maintain panmixia, so greater dispersal of these less-abundant individuals could drive a model of isolation-by-distance as seen throughout other parts of their range [6]. Preliminary genetic data suggests they do not drive such a model (B.M. Gillanders unpublished data), which would indicate either their low abundance, or some other mechanism, restricts gene flow in this cohort. While such considerations are consistent with limited recruitment to the Point Lowly aggregation from southern Spencer Gulf, the genetic barrier also implies dispersal of aggregation-spawned cuttlefish is confined to an area less than 100 km south of Point Lowly.

Oceanographic modeling suggests 70–80% of cuttlefish hatched at Point Lowly in October (the peak timing of cuttlefish hatching) are likely to become confined to the western side of Spencer Gulf by February, some 40–50 km from the aggregation site [26]. Critically, northern Spencer Gulf experiences a slight clockwise circulation of water masses during summer [26], [44], which would make it more energetically expensive for these cuttlefish to travel south (towards southern Spencer Gulf) than to travel north (towards Point Lowly) in the months leading up to the spawning period. With the poor sustained swimming capacity seen for *S. apama* hatchlings, such a scenario could be a major driver of a lack of genetic contribution of Point Lowly cuttlefish to the southern reaches of Spencer Gulf.

## Conclusions

Our estimates of swimming capacity and dispersal potential, coupled with considerations of the behaviour, life-history strategy and oceanography experienced by *S. apama*, are entirely consistent with the striking population divergence observed for this species in Spencer Gulf. Despite protection afforded by a fishing closure, the unique Point Lowly aggregation has experienced drastic declines in number and biomass over the past decade and while the mechanisms for this decline remain unknown, our data suggest recovery of that population is unlikely to be supported by recruits from southern Spencer Gulf. The *S. apama* system provides an example of how a comparison of predicted and observed population connectivity may facilitate a mechanistic understanding of population structuring, which will ultimately improve the ability to predict population change.
